# Effect of weaning age on pregnancy rates in Braford beef heifers bred at 13 months

**DOI:** 10.1590/1984-3143-AR2025-0043

**Published:** 2026-01-16

**Authors:** Maria Eduarda Cocco Dallanóra, José Fernando Piva Lobato, Javier Alexander Bethancourt-Garcia, Mariana Assis Borges, Rangel Fernandes Pacheco, João Restle, Ricardo Zambarda Vaz

**Affiliations:** 1 Departamento de Zootecnia, Universidade Federal de Santa Maria – UFSM, Santa Maria, RS, Brasil; 2 Departamento de Zootecnia, Universidade Federal do Rio Grande do Sul – UFRGS, Porto Alegre, RS, Brasil; 3 Departamento de Zootecnia, Universidade Federal de Lavras, Lavras, MG, Brasil; 4 Departamento de Zootecnia e Ciências Biológicas, Universidade Federal de Santa Maria – UFSM, Palmeira das Missões, RS, Brasil; 5 Departamento de Zootecnia Instituto Federal Farroupilha – IFFar, Frederico Westphalen, RS, Brasil; 6 Departamento de Zootecnia, Universidade Federal de Goiás – UFG, Goiânia, GO, Brasil

**Keywords:** beef cattle, conventional weaning, early weaning, fertility, puberty

## Abstract

The aim of this study was to evaluate the probability of pregnancy in heifers weaned at different ages and bred at 13 to 15 months old. A total of 121 Braford heifers were used, weaned as calves at 77 days (early) or 147 days (conventional) of age. To develop the statistical models of reproductive performance, factors related to the development of the heifers were analyzed. The analysis included a diagnosis of multicollinearity using the Pearson correlation matrix, adjusting the model by means of the Hosmer and Lemeshow test. The response variable, rate of pregnancy, was analysed using the LOGISTIC procedure. Beginning with a weight of 271 kg and an age of 402 days at the start of the breeding season, the pregnancy rates increased by 18.4% and 29.0%, respectively for every 15 kg increase in body weight and 10-day increase in age. However, a reduction of 15 kg in body weight and of 10 days in age reduced the pregnancy rates in the heifers by 15.5% and 22.5%. An increase or reduction of 0.100 kg in the average daily gain between early weaning and conventional weaning represented an increase of 44.6% and a reduction of 30.9% in the chances of pregnancy. Early-weaned heifers require correct nutritional management to allow satisfactory postweaning weight gains so as not to compromise their reproductive performance.

## Introduction

Beef production is facing a period of increasing global demand, driving the need for both male and female calves with enhanced genetic precocity, thereby reducing carbon emissions per unit of meat produced (Barbero et al., 2021). Lactation affects the resumption of cyclicity, particularly in beef females, as it increases nutrient demands for milk production and is often associated with negative energy balance and body condition score loss postpartum.

During the breeding season (BS), the cow is affected by the presence of its calf, since milk production has a direct impact on postpartum anoestrus and increases the calving interval (Vaz and Lobato, 2010c; Orihuela and Galina, 2019). Early weaning, by removing both the suckling stimulus and the physical presence of the calf, contributes to the resumption of cyclicity (Silveira et al., 2019). This effect is further enhanced by the metabolic shift in nutrient partitioning resulting from the cessation of lactation towards the resumption of cyclicity. There is consensus in the literature that during the postweaning period both male calves (Restle et al., 1999a) and female calves (Restle et al., 2009) weaned early have lower performance. As such, early weaning determines a poorer performance until the calf reaches the age of conventional weaning. If properly managed during the post-weaning period, early weaned calves can compensate for this initial disadvantage, exhibiting similar growth and development by 12 months of age (Vaz and Lobato, 2010b; Vaz et al., 2011).

Even with similar development, the different dietary restrictions experienced by the heifers during the various periods of development can alter their fertility (Rosa et al., 2012; Vaz et al., 2012). Reproductive precocity in beef heifers depends, in addition to genetic factors, on the various environmental factors faced during the production cycle (Reis et al., 2023). The complexity inherent to any beef production system makes heifer reproduction a challenging process to manage, especially when grazing, if the key factors that determining reproductive success are not properly quantified (Eloy et al., 2022; Reis et al., 2023; Vaz et al., 2023).

Although early weaning has a positive effect on pregnancy in beef cows (Vaz and Lobato, 2010c; Alforma et al., 2023), the reduced performance of early-weaned heifers could have a negative effect on their reproduction at 13 to 15 months of age (Restle et al., 2009; Vaz and Lobato, 2010b). Our hypothesis was that despite showing similar development at 13 to 15 months, the age at weaning would result in a difference in the growth curves of Braford heifers. Greater or lesser acceleration in body weight during growth may affect the reproductive performance of beef heifers mated early at 13 to15 months (Vaz et al., 2012; Teixeira et al., 2024). However, breeding heifers at 13-15 months of age is not a common practice in southern Brazil. As livestock farming evolves, reducing the age at first breeding is essential for increasing livestock profitability. Therefore, it is necessary to gradually evolve production systems to breed heifers at 13-15 months of age, especially in grazing systems. However, there are challenges in achieving adequate growth and pregnancy rates with these young females. The aim of the present study was to identify growth variables with the potential to predict pregnancy in early- or late-weaned Braford heifers maintained on pasture and submitted to natural breeding between 13 and 15 months of age.

## Methods

All animal handling and procedures were approved by the Ethics Committee for Animal Use (CEUA) of the Federal University of Santa Maria under number 2388280122.

### Location

The experiment was carried out on private property in the district of Itaqui in the south of Brazil, at 29°12’S and 55°36’W. The terrain in the region is undulating with deep soils. According to the Köppen classification, the climate is subtropical (Alvares et al., 2013).

### Animals

The experiment included 121 Braford heifers, weaned earlier (EW - 77 days of age - range of 68 to 90 days) or Later (LW - 147 days of age - range of 128 to 176 days), breed between 13 and 15 months of age. Of the 121 heifers, 77 belonged to the EW group and 44 to the LW group. This numerical difference resulted from adverse forage conditions caused by drought, which led to a management decision to wean more calves in order to better accommodate the breeding herd. Nevertheless, criteria such as calving season and cow age were maintained in both the EW and LW groups.

The heifers were born to primiparous and second-calving cows (three and four years old, respectively), with calvings occurring between September 10st and November 10st. As calving occurred, the cows and their calves were allocated to treatments, aiming for an even distribution of cow age and calving date.

### Nutritional/diet management

**Figure 1:**
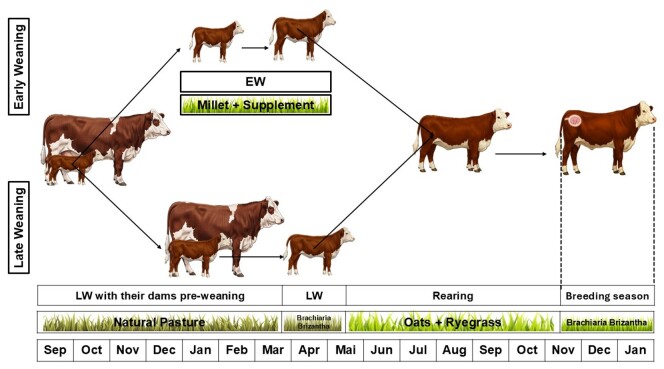
Timeline of key events and procedures during the development of the heifers (Calving-end of the breeding season).

The calves, while still with their dams before weaning, were kept on natural pasture at a stocking rate of 320 kg of body weight/ha. Following weaning, whether EW or LW, the calves were placed in a pen for 10 days, with supplementation from day one and short periods of hourly grazing beginning on the fourth day after weaning. EW heifers were weaned in December, January and February. Weaning’s were carried out every 10 days, with calves being weaned at around 75 days of age. Following the stressful weaning period, the EW calves were kept during the summer and early autumn (December–March) on a pasture of cultivated millet (*Pennisetum americanum*) at a stocking rate of eight calves/ha. The LW calves were similarly kept in a pen for 10 days and then joined the EW calves on the millet pasture. During April, all the calves grazed on Brachiaria Brizantha (*Brachiaria brizantha* ‘Marandu’). Heifers from the EL group were weaned at beginning of March (Figure 1).

During the postweaning period, from the first 10 days in the pen to the start of the winter pasture (May), all of the calves received a supplement at 1% of body weight, of 18% crude protein and 75% total digestible nutrients. The feed was formulated on the property using soybean meal, soybean hulls, wheat bran, whole rice bran, common salt, calcitic limestone and a mineral mixture (Figure 1).

During the winter and spring (May-November), the calves grazed on oats (*Avena strigosa*) and ryegrass (*Lolium multiflorum* Lam) at a stocking rate of five animals/ha until the start of the BS in November, which was carried out on natural pasture (Figure 1). During the experiment, the calves had free access to a mineral mixture that included 60 ppm phosphorus; during the BS, this was increased to 80 ppm phosphorus. Both mixtures comprised common salt and dicalcium orthophosphate, which were mixed on the property.

The available forage was estimated using the comparative method, with five cuts of 0.25 m2 made close to the ground and scored from one (low availability) to five (maximum availability). A further 20 random visual assessments were then made and scored based on the above assessments.

### Animal measurements (weights)

All heifers were weighed at birth, at EW or LW, at the start and end of the period of winter pasture, and at the start and end of the BS at 13 to 15 months of age (November 1st to January 11st). The calves were also weighed every 28 days to adjust the stocking rates of the different pastures as the heifers developed. The daily weight gain was calculated as the difference in weight between each weighing divided by the interval between weighings. The body condition score (BCS) was assessed when weighing, assigning values from 1 to 5, where 1 = very lean and 5 = very fat (Lalman and Stein, 2024).

### Reproductive management

The BS ocurred during the spring and summer (November-January), when heifers were between 13 and 15 months old and lasted for 72 days. All heifers entered the breeding season on the same starting date, with the only variation being their ages and weaning ages. Natural breeding was used as the method of reproduction, employing bulls previously approved for libido and by andrological examination. The bull/heifer ratio was 1:25, giving a total of six bulls. Five bulls were kept with the group of heifers, with the bull with the worst body condition resting for 10 days. Pregnancy was diagnosed by ultrasonography carried out 30 days after the end of the BS.

### Independent or predictor variables

Evaluating each heifer during growth, the following variables were determined as predictors of pregnancy for mating performed on average at 13 to 15 months of age: body weight at birth, at weaning, at the start and end of the production cycle on the winter-spring pastures, and at the start and end of the BS at 13 to 15 months of age, as well as the age of the heifers at the start of the BS. The body condition score was evaluated at each weighing except at birth, as well as the average daily gain between each period. To evaluate the group of heifers, regardless of weaning age, the lactation period in days was also considered.

### Statistical analysis

The SAS statistical package (Statistical Analysis System, v 9.2) was used to develop the models and carry out the statistical analysis. Initially, the pregnancy rate parameter for cows and their heifers, based on different weaning ages, was analyzed using the Chi-Square test at a 5% significance level.

For heifers, the response variable, rate of pregnancy, was determined by whether the heifer was pregnant or not, and was analysed using the LOGISTIC procedure. Three models were fitted, the first including all heifers regardless of their weaning age, the second for EW heifers, and the third for LW heifers.

Multicollinearity between the predictor variables was diagnosed using the Pearson correlation matrix (Khalaf and Iguernane, 2016), selecting the most significant variables to build the model that best explained the pregnancy of the heifers. These were tested using multiple linear and quadratic regression models (Hosmer et al., 2013). In selecting the best model, the Hosmer and Lemeshow goodness-of-fit test was used, considering p > 0.30 (Hosmer et al., 2013).

Three different models were fitted: one covering all heifers regardless of their weaning age and two specifically for the categories of EW and LW heifers. After fitting the models, the βi parameters were estimated, followed by tests to verify the significance of the independent variables and their relationship with the probability of pregnancy. To assess the quality of the adjusted model and the relevance of the individual parameters, the Wald test and the score test were applied.

The adjusted multiple regression equations represented the overall rate of pregnancy, the rate for early-weaned heifers, and the rate for LW heifers (1), providing a detailed analysis of the impact of the variables in each group.


$$Pi=\;\frac{exp\left(yi\right)}{1+\exp\left(yi\right)}={\left[1+\exp\left(yi\right)\right]}^{-1}$$ (1)

where Pi is the probability of the i-th heifer being diagnosed as pregnant.

For the overall pregnancy of the heifers regardless of weaning age, Pi was established using the following Equation 2:


yi=µ+β1X1i+β2X2i+β3X3i+εi (2)

where µ is a constant; X1i, the weight at the start of the BS of the i-th heifer (kg); X2i, the average daily weight gain from EW or LW of the i-th heifer (kg); X3i, the age at the start of the BS of the i-th heifer (days); εi, random error associated with the i-th heifer.

For EW heifers, the probability of the i-th heifer being diagnosed as pregnant was established using the following Equation 3:


yi=µ+β1X1i+β2X2i+εi (3)

where µ is a constant; X1i, the weight at the start of the BS of the i-th heifer (kg); X2i, the average daily weight gain from EW to LW of the i-th heifer (kg); εi, random error associated with the i-th heifer.

For LW heifers, the chance of the i-th heifer being diagnosed as pregnant was established using the following Equation 4:


yi=µ+β1X1i+β2X2i+εi (4)

where µ is a constant; X1i, the weight at the start of the BS of the i-th heifer (kg); X2i, the average daily weight gain from birth to conventional weaning of the i-th heifer (kg); εi, random error associated with the i-th heifer.

Regardless of the model, the odds ratio, estimated by OR=exp (bk), was used to interpret the coefficients, where OR is the ratio of the proportions for two possible outcomes, i.e. the ratio between success (πj) and failure (1 - πj) of the heifer becoming pregnant. The odds ratios were based on the mean denominator of the data set for each model. The units of change for the regressor variables with the potential to increase or decrease the pregnancy of the heifers were 15 kilograms for the weight at the start of the BS, 10 (ten) days for the age at the start of the BS, and 0.100 kg for the average daily gain from EW to LW.

## Results

The average pregnancy rate of early weaned cows was 86.34%, higher (P<0.05) than that of late weaned cows, at 55.45%. In heifers, the average pregnancy rate was similar between weaning ages (Table 1).

**Table 1:** Beef cow pregnancy rates during the breeding season according to the weaning age of their calves.

Weaning age	Total	Pregnant	Not pregnant	Pregnancy rates
*Cows*				
Early weaning	77	65	12	84.4^a^
Late weaning	44	23	21	52.3^b^
*Heifers*				
Early weaning	77	41	36	53.2^a^
Late weaning	44	32	12	72.7^a^

a,b Within-animal category, difference (P<0.05) by the Chi-Square test.

Eight models were tested for each class of animal to create the final models, one unifying all heifers regardless of weaning age, the second for EW heifers, and the third for LW heifers (Table 2). The models were built by introducing and removing variables for predicting pregnancy at 13 to 15 months of age, arriving at the model that best explains the pregnancy of the heifers.

**Table 2:** Models tested to build regression equations for variables that resulted in pregnancy in heifers mated at 13/15 months of age.

Model	Unified – Both weaning ages		Early weaning		Late weaning	
Model 1	WSBS + DGEC + ASBS	0.96	ASBS + DGEC	0.93	GBCW + WSBS	0.93
Model 2	ASBS + WSBS + DGBS	0.90	ASBS + GBEW	0.92	GBCW + WCW	0.91
Model 3	ASBS + WSBS + WCW + SUCKLE	0.62	ASBS + GBEW + DGEC	0.83	GBCW + WEBS + WCW	0.81
Model 4	SUCKLE + WCW	0.59	ASBS + GBEW + WSBS	0.74	GBCW + WEBS	0.80
Model 5	WCW + DGEC	0.58	GBEW + PSP	0.73	GBEW + BCSEBS	0.73
Model 6	DGEC + WCW + SUCKLE	0.58	ASBS + GBEW + WEBS	0.69	GBCW + PSP + ASBS	0.69
Model 7	DGEC + WCW + SUCKLE	0.58	WSBS + GBEW	0.63	GBEW + WEW + DGEC	0.62
Model 8	ASBS + DGBS	0.54	GBCW + ASBS + WEBS	0.61	GBEW + DGEC + ASBS	0.60

ASBS: age of the heifer at the start of the BS; DGBS: daily weight gain during the BS; DGEC: average daily gain of the calf from early weaning to conventional weaning; SUCKLE: number of calf suckling days; WSBS: weight of the heifer at the start of the BS; WCW: weight of the calf at conventional weaning; GBEW: daily weight gain from birth to early weaning; WEW: weight of the calf at early weaning; PSP: body weight of the calf at the start of the winter-spring pastures; WEBS: body weight of the heifer at the end of the BS; GBCW: daily weight gain from birth to conventional weaning; BCSEBS: body condition score of the heifer at the end of the BS.

This model explained 96%, 93% and 93% of the pregnancy of Braford heifers for the combined, EW and LW groups, respectively. The better-fitting models often exclude variables that, albeit determinants of pregnancy, are not selected as they are highly correlated with another variable that better explains the pregnancy of the heifers. For the combined evaluation regardless of weaning age, the suckling period when the heifers were calves, explained 62% of the pregnancy. However, the weight gain of the calves during the suckling period between the weaning ages was included in the final model, explaining 96% of the pregnancy.

The descriptive analysis of the heifer data showed an average rate of pregnancy of 60% for heifers mated at 13 to 15 months of age (Table 3). Pregnancy rate was 37.7% higher in LW heifers (73%) compared to EW heifers (53%) (Table 3).

**Table 3:** Descriptive analysis heifers mated at 13 to15 months of age and subjected to early or conventional weaning when calves.

Variable	N	Mean	SD	Min	Max	N	Mean	SD	Min	Max	N	Mean	SD	Min	Max	P-value
	*All heifers*					*Early weaned heifers*					*Late-weaned heifers*					
*Weight, kg*																
At EW	121	81.4	13.1	59.0	123.0	77	81.1	12.8	60.0	120.0	44	82.0	13.6	59.0	123.0	NS
At CW	121	118.5	20.0	79.0	178.0	77	112.7	19.7	80.0	178.0	44	127.8	20.3	92.0	171.0	**
Start of WP	121	144.7	25.5	101.0	221.0	77	145.2	26.3	102.0	221.0	44	143.7	24.2	101.0	213.0	NS
Start of BS (13 months)	121	271.0	31.9	165.0	361.0	77	271.7	31.6	198.0	361.0	44	269.8	32.9	165.0	321.0	NS
End of BS (15 months)	121	297.3	34.7	176.0	390.0	77	296.7	33.7	205.0	390.0	44	298.2	36.6	176.0	353.0	NS
*Average daily gain, kg*																
From birth to EW	121	0.67	0.13	0.29	0.97	77	0.67	0.12	0.34	0.96	44	0.65	0.15	0.29	0.97	NS
From EW to CW	121	0.53	0.18	0.16	1.06	77	0.51	0.16	0.16	0.90	44	0.58	0.19	0.19	1.06	*
From birth to CW	121	0.60	0.11	0.34	0.90	77	0.59	0.10	0.38	0.87	44	0.62	0.13	0.34	0.90	NS
During BS (13-15 months)	121	0.39	0.15	-0.07	0.75	77	0.37	0.15	-0.07	0.64	44	0.43	0.15	0.15	0.75	NS
*Body condition score, points*																
Start of BS (13 months)	121	3.87	0.40	3.00	4.90	77	3.84	0.41	3.00	4.90	44	3.94	0.37	3.00	4.50	NS
End of BS (15 months)	121	3.84	0.55	2.80	4.80	77	3.78	0.56	3.00	4.80	44	3.95	0.50	2.80	4.70	*
*Ages of heifers*																
Age at early weaning, days	121	80	3.21	58	111	77	77	3.53	58	111	44	81	3.71	59	110	NS
Age at late weaning, days	121	147	4,18	128	182	77	146	4.15	128	182	44	147	4.24	130	180	NS
Age at start of BS, days	121	402	17.4	358	433	77	401	17.0	358	433	44	403	18.4	362	432	NS
*Reproductive performance*																
Pregnancy, %	121	0.60	0.49	0.00	1.00	77	0.53	0.50	0.00	1.00	44	0.73	0.45	0.00	1.00	NS

SD: Standard Deviation; Min: Minimum; Max: Maximum; EW: Early Weaning; CW: Conventional Weaning; WP: Winter Period; BS: Breeding season; P-values: Comparative between early weaned and late weaned; NS: Not significant. *P<0.05; **P<0.01.

Regardless of the weaning age of the heifers, the variables that promoted pregnancy were weight and age at the start of the BS at 13 to 15 months of age, and the gain in body weight between the average ages of EW and LW (Table 4). Our results showed that the heifers weighed 271 kg at the start of the BS and were 402 days old. However, for each 15 kg increase in body weight and 10-day increase in age, the probability of pregnancy increased by 18.4% and 29.0%, respectively, while a 15 kg reduction in body weight and a 10-day reduction in age reduced the chances of pregnancy by 15.5% and 22.5%.

**Table 4:** Estimates of the chances of an increase or decrease in pregnancy in heifers evaluated as a single group and by weaning age, mated when 13/15 months old, based on the regressor variables.

Variable	Estimate	95% CL	Increase		Reduction		*P-value*
			Units	OR	Units	*OR*	
*EW and LW groups Combined*							
Intercept	-14.735	-24.970 a -4.495					0.004
WSBS, kg	0.011	-0.005 a 0.028	15	1.184	-15	0.845	0.199
ASBS, days	0.025	-0.002 a 0.053	10	1.290	-10	0.775	0.074
DGEC, kg	3.691	0.890 a 6.492	0.100	1.446	-0.100	0.691	0.009
*Early-weaned heifers*							
Intercept	-21.973	-35.802 a -8.144					0.001
ASBS, days	0.050	0.016 a 0.084	10	1.731	- 10	0.366	0.003
DGEC, kg	3.916	0.471 a 7.362	0.100	1.479	- 0.100	0.676	0.025
*Late weaned heifers*							
Intercept	-11.424	-20.430 a -2.417					0.0129
GBCW, kg	8.519	0.516 a 16.520	0.100	2.344	- 0.100	0.427	0.0369
WSBS, kg	0.028	-0.0004 a 0.055	15	1.416	- 15	0.660	0.0401

CL: confidence limits; OR: odds ratio; EW: Early Weaning; LW: Late weaning; WSBS: weight at the start of the BS; ASBS: age at the start of the BS; DGEC: average daily gain of the calf from early weaning to conventional weaning; GBCW: Gain from birth to conventional weaning.

With an average daily gain of 0.530 kg between EW and LW (77 and 147 days of age, respectively), an increase of 0.100 kg represents a 44.6% increase in the chances of pregnancy, while a reduction of 0.100 kg during the same period affords a 30.9% reduction in the chances of pregnancy in heifers mated at 13 to 15 months of age, regardless of weaning age.

For EW heifers, the regressor variables that influenced pregnancy at 13 to 15 months of age were age at the start of the BS and average daily gain between EW and LW. Every additional 10 days in the age of the heifers at the start of the BS resulted in an increase of 73.1%, while a 10-day reduction in age resulted in a 73.4% reduction in pregnancy. As for performance between weaning ages, an increase or decrease of 0.100 kg during the period between EW and LW afforded an increase of 47.9% and decrease of 32.4%, respectively, in the pregnancy rate of EW heifers.

In the LW heifers, with their longer suckling period, preweaning performance was a determining factor in reproductive success. Starting from an average daily gain of 0.580 kg, an increase of 0.100 kg improved the probability of pregnancy by 134%. In contrast, a loss of 0.100 kg resulted in a 57.3% reduction in the pregnancy rate of heifers mated at 13 to 15 months of age. Heavier heifers at the start of the BS were more fertile, where for each 15 kg above or below the average weight of 269.8 kg there was a respective 41.6% increase or 34.0% reduction in the pregnancy of LW heifers mated at 13 to 15 months of age.

## Discussion

The better reproductive performance of cows weaned at 77 days post-calving is partially due to their higher weight gain in the period between the two weaning ages and during the breeding season, resulting in better body condition. This shows that early weaning, by removing both the suckling stimulus and the physical presence of the calf, contributes to the resumption of cyclicity (Silveira et al., 2019). This effect is further enhanced by the metabolic shift in nutrient partitioning resulting from the cessation of lactation towards the resumption of cyclicity (Vaz and Lobato, 2010c).

Pregnancy in beef cattle depends on several factors. When evaluating reproductive performance in beef heifers, several factors can influence the reproductive success of a herd. The use of robust statistical tools such as the LOGISTIC procedure, together with goodness-of-fit tests, can ensure reliability and accuracy in identifying determining factors of pregnancy. As such, it is necessary and correct to use multivariate statistical analysis, which can determine the main influencing variables, as well as predict an increase or decrease in the rate of pregnancy from an increase or decrease in these variables. The literature shows the importance of these analyses to understanding the complexities involved in the reproductive performance of beef cows (Pacheco et al., 2022; Reis et al., 2023; Vaz et al., 2023) but, to our knowledge, no studies have evaluated the effect of weaning age on the reproductive performance of beef heifers using the statistical approach applied in the present study. The advantage of these analyses lies in determining multicollinearity diagnosed by means of the Pearson correlation matrix, where such factors as weaning weight may present a high correlation with the type of management adopted (early or late). This led us to conduct a separate study based on the weaning age of heifers, to verify the effect of weaning age on pregnancy in heifers mated at 13 to 15 months old.

Female calves subjected to early weaning usually have a lower weight than those weaned conventionally, due to the smaller average daily gains immediately after weaning (Vaz and Lobato, 2010b). The smaller weight gains of EW calves are due to stress and adapting to solid food, while those LW show larger gains due to the continued consumption of maternal milk (Restle et al., 1999a, 2009; Teixeira et al., 2024). However, the smaller weight gain caused by early weaning should not be allowed to harm the growth or future performance of the female (Orihuela and Galina, 2019).

This study clearly demonstrates the importance of weight gain in heifers during the period between the two weaning ages while they are still calves, where this characteristic was shown to promote pregnancy at 13 to 15 months of age for the heifers evaluated as a single group and for the group of EW heifers. Although early weaning favours the reproductive performance of the cows (Vaz and Lobato, 2010c), the performance of the calves following the withdrawal of maternal milk must not be impaired. Several studies show that there is no difference in performance in terms of weight, body condition score or reproduction, in animals at 12 months of age whether subjected or not to early weaning (Restle et al., 1999a, 2009; Vaz and Lobato, 2010b). However, we did not find in the literature, post-weaning development being evaluated by regression to demonstrate the effect on the reproductive performance of heifers mated at 13 to 15 months, and assessing the nutritional level imposed on the calves during the postweaning period and their effect when the calves are exposed to reproduction at a young age. Our results show that the increase in weight gain following EW favours a significant increase in pregnancy when heifers are mated at 13 to 15 months of age, where poor performance during the postweaning period is detrimental to pregnancy. This shows that the reproductive performance of the heifers is directly related to nutrition and the environment, as well as the management practices adopted throughout the animals’ development (Eloy et al., 2022).

Therefore, EW should not be evaluated solely on its benefits for the pregnancy of the cow, since calves subjected to EW must perform satisfactorily to avoid poor reproductive performance at 13 to 15 months of age. Our results showed that EW was associated with unsatisfactory weight gain which was a limiting factor for successful reproduction in heifers with first mated at 13 to 15 months old. On the other hand, Silveira et al. (2019) showed that heifers weaned early could reach suitable weights for puberty as long as they were subjected to adequate nutritional programs with no restrictions that might delay puberty and reproductive success.

The average pregnancy rate of 60% for the heifers, regardless of weaning age, is similar to that found in the Brazilian production sector, but remains below the ideal rates of reproduction. However, the 73% rate of pregnancy for LW heifers is similar to that of well-structured production systems. This difference suggests that there is room for improvement, where greater weight at the start of the BS determines a higher rate of pregnancy for the heifers as a single group and for those LW. Understanding the variations in pregnancy rate in different production systems is crucial for adapting management strategies to the specific conditions of each system. The greatest contribution of weight at the start of the BS was seen in the group of LW heifers, showing that, for reproductive success, animals need to be of good weight at weaning and at the start of breeding. Therefore, in production systems where EW is not necessary to improve reproduction of the cows, better calf development is essential for them to become pregnant. Although there was no difference in body weight at the beginning of the BS (Table 3), the LW heifers were superior during pregnancy. This fact can be explained by the nutritional restriction of the EW heifers during a crucial phase for intensive production systems. Furthermore, the average weights at the beginning and end of the BS can be considered low for the type of animal studied, a factor that may be decisive in reproductive success.

Working with Braford heifers and Charolais-Nellore crosses, and stratifying the herds into weight classes, Vaz and Lobato (2010a) and Vaz et al. (2012), respectively, found higher rates of pregnancy for heifers in the heaviest classes, with rates greater than 90% at 13 to 15 months for heifers weighing over 300 kg in both studies. In retrospective studies, Rocha and Lobato (2002a, b) and Landarin et al. (2016) found that heifers pregnant at 13 to 15 months of age were the heaviest at the start and end of the BS. Greater heifer weight can therefore be a successful tool for selecting animals with a greater chance of pregnancy when mated early.

A higher age at the start of the BS proved to be important for pregnancy at 13 to 15 months of age in the heifers when evaluated as a single group i.e. irrespective of weaning age, and for the group of EW calves. This shows the importance of early births during the calving season, where the earlier a calf is born, the greater its chance of becoming pregnant when mating at a younger age, compared to calves born later, identified in the difference in pregnancy rate in the present study that favoured animals born earlier in the season. These results agree with those of Vaz et al. (2020), who found that heifers mated at 13/15 months of age under similar management and born in the same calving season, had pregnancy rates of 70% or 43% when the average age difference was only 30 days. In a retrospective analysis of their studies, Rocha and Lobato (2002ab) and Vaz et al. (2012) found that pregnant heifers in intensive production systems, such as in the present study, started the BS at a greater age compared with those that failed to get pregnant. Our results, although not evaluating the onset of puberty, together with the literature, highlight the importance for puberty of the interaction between weight and age, with puberty determined by both genetic and environmental factors (Restle et al., 1999b), where a heifer enters puberty upon reaching its target weight, which in turn depends on the nutritional management of the herd. Eloy et al. (2022) point out that factors such as the quality of the pasture, supplements, and management practices play a crucial role in controlling precocious puberty. Furthermore, the interaction between weight and age suggests that individualised strategies, adapted to the specific conditions of each production system, can maximise reproductive results (Silveira et al., 2019).

For LW heifers, weight gain from birth to weaning was a determining factor of increased pregnancy. Preweaning weight gain has a direct impact on the weaning weight of the calves. This increase in preweaning weight reflects the importance of suckling to the reproductive performance of beef heifers, favouring increased pregnancy when mating at 13 to 15 months of age. When a calf is weaned at 147 days and mated at 13 to 15 months, it spends 37% of its life with its mother, with the calf highly dependent on the milk produced by the cow. Evaluating high and low nutritional levels pre- and postweaning, Vaz et al. (2012) found an average pregnancy rate of 87% and 54%, respectively, for high and low nutritional levels during the preweaning period, highlighting the importance of the suckling period in more-intensive production systems, where the age of the heifers when first mated is reduced to 13-15 months.

The results of this study have practical implications for the reproductive management of beef heifers mated at 13 to 15 months of age. The adoption of EW should be planned, taking into account both the reproductive benefits for the cow and the proper development of the calves in relation to the goals of the production system. It is therefore important to adopt an integrated approach that combines nutrition and management adapted to the specific characteristics of each production system, with sustainable and economically viable strategies to maximise the productivity and reproductive efficiency of the herd.

Our study, although with satisfactory results, has some weaknesses that should be highlighted. First, for future studies, the number of animals should be increased to provide greater reliability in the results. Another determining factor would be the increase in animal weights at the beginning of the reproductive period, which were low for the type of animal studied. To further strengthen the data, the evaluation of physiological indicators would be a very enlightening alternative to the results.

## Conclusions

For the reproductive success of beef heifers at 13 to 15 months of age, it is important that they are born to cows that calved early in the calving season, show good body development and start the breeding season with sufficient weight and physiological maturity. Early-weaned heifers require correct nutritional management to allow satisfactory postweaning weight gains so as not to compromise their reproductive performance. Furthermore, late weaned heifers need to show good preweaning performance to ensure reproductive success at 13 to 15 months of age.

## Data Availability

Please contact author for data requests.

## References

[B001] Alforma AMP, Pereira GR, da Rocha MK, Teixeira OS, Oliveira MCM, Lima JA, Cumbe TA, Barcellos JOJ (2023). Influence of weaning management at 30, 75 and 180 days of age on non-esterified fatty acids and reproductive performance in beef cows. J Anim Physiol Anim Nutr.

[B002] Alvares CA, Stape JL, Sentelhas PC, Moraes GJL, Sparovek G (2013). Köppen’s climate classification map for Brazil. Meteorol Z.

[B003] Barbero RP, Ribeiro ACC, Moura AM, Longhini VZ, Mattos TFA, Barbero MMD (2021). Production potential of beef cattle in tropical pastures: a review. Cienc Anim Bras.

[B004] Eloy LR, Bremm C, Lobato JFP, Pötter L, Laca EA (2022). Direct and indirect nutritional factors that determine reproductive performance of heifer and primiparous cows. PLoS One.

[B005] Hosmer D, Lemeshow WS, Sturdivant RX (2013). Applied logistic regression..

[B006] Khalaf G, Iguernane M (2016). Multicollinearity and a ridge parameter estimation approach. J Mod Appl Stat Methods.

[B007] Lalman D, Stein D (2024). Body condition scoring of cows.

[B008] Landarin CM, Lobato JFP, Tarouco AK, Tarouco JU, Eloy LR, Pötter L, Rosa AAG (2016). Growth and reproductive performance of 14- to 15-month-old Hereford heifers. Rev Bras Zootec.

[B009] Orihuela A, Galina CS (2019). Effects of separation of cows and calves on reproductive performance and animal welfare in tropical beef cattle. Animals.

[B010] Pacheco RF, Vaz RZ, Restle J, Silveira MF, Cerdotes L, Teixeira JS, Milani L, , Pacheco PS (2022). Probability of pregnancy in beef cows with early-weaned calves. Livest Sci.

[B011] Reis NP, Lobato JFP, Restle J, Pacheco RF, Nuñez AJC, Sartori DBS, Vaz RZ (2023). Effect of the performance, calving date and lactation period on the Probability of pregnancy in beef cows. Sci Agric.

[B012] Restle J, Polli VA, Alves  DC, Senna DB, Vaz RZ, Bernardes RACL, Silva JHS (1999). Desenvolvimento de bovinos de corte de diferentes grupos genéticos desmamados aos 3 ou 7 meses de idade. Rev Bras Zootec.

[B013] Restle J, Polli VA, Senna DB (1999). Efeito de grupo genético e heterose sobre a idade e peso à puberdade e sobre o desempenho reprodutivo de novilhas de corte. Pesqui Agropecu Bras.

[B014] Restle J, Vaz RZ, Pascoal LL, Alves DC, Vaz FN, Segabinazzi LR (2009). Desenvolvimento e desempenho reprodutivo de novilhas de corte submetidas a diferentes idades de desmame. Cienc Anim Bras.

[B015] Rocha MG, Lobato JFP (2002). Avaliação do desempenho reprodutivo de novilhas de corte primíparas aos dois de idade. Rev Bras Zootec.

[B016] Rocha MG, Lobato JFP (2002). Sistemas de alimentação pós-desmama de bezerras para acasalamento com 14/15 meses de idade. Rev Bras Zootec.

[B017] Rosa AAG, Vaz RZ, Lobato JFP (2012). Natural and improved pastures on growth and reproductive performance of Hereford heifers. Rev Bras Zootec.

[B018] Silveira MF, Restle J, Brondani IL, Machado DS, Pacheco RF, Argenta FM, Silva VS, Hoffmann F (2019). Effect of age and genetic group on the development of calves weaned at 63 days until one year of age. Semina: Ciênc Agrár.

[B019] Teixeira OS, Camargo VA, Rocha MK, Alforma AMP, Sartori ED, Rosa YM, Pérez-Atehortúa M, McManus C, Barcellos JOJ (2024). Three ages at weaning in beef calves: implications on performance and development. Rev Bras Zootec.

[B020] Vaz RZ, Lobato JFP (2010). Efeito da idade de desmame no desempenho reprodutivo de novilhas de corte expostas à reprodução aos 13/15 meses de idade. Rev Bras Zootec.

[B021] Vaz RZ, Lobato JFP (2010). Efeito da idade do desmame no desenvolvimento de novilhas de corte até os 14/15 meses de idade. Rev Bras Zootec.

[B022] Vaz RZ, Lobato JFP (2010). Effects of the weaning age of calves on somatic development and on reproductive performance of beef cows. Rev Bras Zootec.

[B023] Vaz RZ, Lobato JFP, Vaz FN, Restle J, Pascoal LL (2011). Características da carcaça de novilhos desmamados aos 91 ou 160 dias terminados em pastagem e abatidos aos dezesseis meses com diferentes pesos. Cienc Anim Bras.

[B024] Vaz RZ, Restle J, Pacheco PS, Vaz FN, Pascoal LL, Vaz MB (2012). Ganho de peso pré e pós-desmame no desempenho reprodutivo de novilhas de corte aos quatorze meses de idade. Cienc Anim Bras.

[B025] Vaz RZ, Lobato JFP, Restle J, Costa PT, Eloy LR, Ferreira OGL, Costa JLB (2020). Calving month and calf sex on the production and efficiency of herds. Cienc Anim Bras.

[B026] Vaz RZ, Lobato JFP, Bethancourt-Garcia JA, Pacheco RF, Reis NP, Sartori DBS, Jappe SA, Restle J (2023). Environmental factors on the probability of pregnancy in early or conventionally weaned beef cows. Anim Reprod.

